# Infectious Pancreatic Necrosis Virus in Scottish Atlantic Salmon Farms**,** 1996**–**2001

**DOI:** 10.3201/eid0904.020311

**Published:** 2003-04

**Authors:** Alexander G. Murray, Corina D. Busby, David W. Bruno

**Affiliations:** * Fisheries Research Services Marine Laboratory, Victoria Road, Aberdeen, Scotland; † Department of Zoology, University of Aberdeen, Aberdeen, Scotland

**Keywords:** infectious pancreatic necrosis virus, salmon, aquaculture, surveillance, Scotland, research

## Abstract

The rapid growth of aquaculture has provided opportunities for the emergence of diseases. Programs designed to monitor these pathogens are useful for analysis of regional variation and trends, provided methods are standardized. Data from an official monitoring program were used to analyze the emergence of infectious pancreatic necrosis virus in Scottish salmon farms from 1996 to 2001. An annual increase in the prevalence of this virus was found in saltwater (10%) and freshwater sites (2% to 3%), with a much faster increase (6.5%) in Shetland’s freshwater sites. No significant increase in the virus was detected in the marine farms of southern mainland Scotland. However, the virus had become very prevalent at marine sites and was almost ubiquitous in Shetland by 2001, and thus the prevalence of this virus at marine sites may be underestimated. Because several diseases have emerged or are emerging in fish farming, aquaculture surveillance programs represent a rich potential source of data on emerging diseases.

The growth of the aquaculture industry provides a rich environment in which to study emergence of pathogens (*1*,*2*). New fish species are being cultivated, and production has risen rapidly. For example, from 1996 to 2001, Scottish farmed salmon production doubled to 158,000 metric tons (*3*). Shipping is extensive, and stock movement between farms is substantial (*4*). Thus, both host populations and contact opportunities are high, the ideal theoretical conditions for disease transmission (*5*). Indeed, an array of pathogens that affect farmed salmon have emerged (*6*).

Because of the disease threat, official (*6*,*7*) and industry (*8*) surveillance for pathogens is extensive. This surveillance is designed to find pathogens, and thus may bias estimates of true pathogen prevalence. Sampling may be biased to farms perceived to be at risk or to specific times of year. The number of fish in a sample may vary. Similarly, sampling methods have detection limits, which may result in substantial numbers of asymptomatic infections being undetected (*9*,*10*). Nevertheless, a continuous centrally organized national survey exists for pathogens in an environment where pathogens are emerging. This survey has the potential to throw detailed light on patterns of pathogen emergence, even if absolute prevalence may be biased.

Infectious pancreatic necrosis (IPN) is considered the most serious viral disease in terms of its impact on Atlantic salmon (*Salmo salar*) production in the European Union (*11*); the disease occurs on four continents (*7*). The problem is increasing as the disease continues to emerge (*12*). IPN is caused by the *Infectious pancreatic necrosis virus* (IPNV; genus *Aquabirnavirus*) (*13*,*14*). Presence of IPNV is a necessary, but not sufficient, condition for IPN; disease also depends upon viral strain (*15*), environment (*12*), and age of the fish (*12*,*13*). In nondiseased fish, IPNV may be present in small quantities (*13*,*16*); virus in such carrier fish may be difficult to detect, which leads to underreporting (*9*,*10*).

IPN has been known for decades. It was first described in North America in freshwater trout in the 1950s (*17*), although indications of the disease were reported in 1940 (*16*). IPN was first reported in the United Kingdom in 1971 (*18*) in rainbow trout, *Oncorhynchus mykiss,* a North American species. Early reports of IPN in salmonids were associated with deaths of fry (recently hatched fishes) in freshwater hatcheries (*13*), but since the 1980s, deaths have also been increasingly reported among smolts (2-year-old salmon) shortly after transfer to seawater (*15*,*12*). IPNV-positive salmon smolts are estimated to have a mortality rate five times higher than IPNV-negative smolts (*14*). IPN is also associated with less easily quantified losses such as loss of appetite and therefore of production (*19*).

## Methods

**Figure 1:**
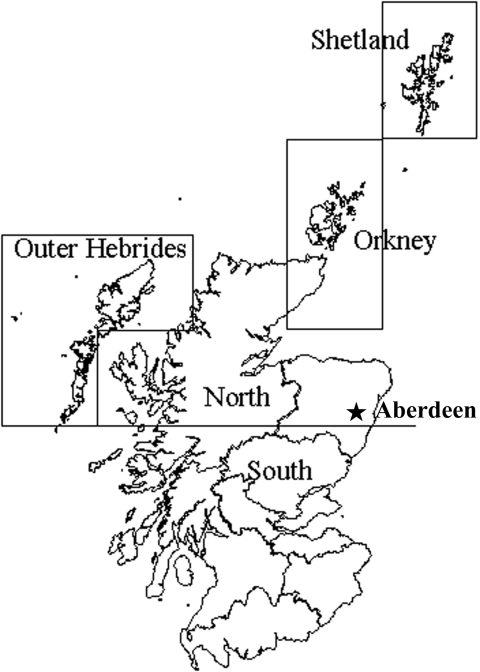
Salmon production regions in Scotland, including the city of Aberdeen, the site of the FRS Marine Laboratory, where fish health inspectors and virologists are based.

Fisheries Research Services (FRS) fisheries inspectors have sampled fish from throughout Scotland for pathogens, including IPNV, for many years. The inspectorate is based in Aberdeen (Figure 1), distant from salmon farming areas on the west coast and Northern Isles (*3*). This central organization is important for the sampling structure because the same persons collected samples from different regions using standard operating procedures, and a single laboratory then processed these samples.

The IPNV diagnosis used cell culture on Chinook salmon embryo cell lines in plates containing 24 wells (*7*). Virus identification was undertaken with enzyme-linked immunosorbent assay (*7*). The standard sample from 30 fish was divided into six pools, each containing material from five kidneys; occasionally 30 pools from individual kidneys were sampled. However, some samples did not contain 30 fish, depending upon opportunity, and varied from 1 to 150 fish kidneys; pool size also varied. Any positive pool generated a positive result at the sample level. Because samples with smaller numbers of fish are less likely to contain virus than are large ones (*20*), IPNV may be missed in small samples. In addition, limits of test sensitivity mean that fish with low IPNV titers may escape detection (*9*,*10*), although if samples are large enough to contain several infected fish, test sensitivity is likely to be less important in detecting IPNV at the sample level (*20*). Small samples from biased locations and test detection limits mean that errors may exist in the absolute prevalence estimates, but with standard methods and centralized personnel, trends and relative regional prevalence can be determined.

FRS scientists have been testing for IPNV since the 1970s (*18*); standard methods approved by the Office International des Epizooties (*7*) were used through the 1990s. In freshwater, sonication has been applied to tissue homogenate since 1993 to disrupt cells and release virus; in saltwater, this process was not begun until 2001. Thus, sensitivity of detection may have been slightly improved for marine samples in the last year and was generally better in freshwater.

Data available for 1996–2001 have been used to find underlying patterns in the distribution of prevalence of IPNV in Scottish salmon farms. Fish were sampled at all stages of the production cycle, although many more samples were collected in summer than in winter, leading to potential biases. Also, inspectors preferentially take moribund fish because these are of most concern and could indicate a disease outbreak. Most sites were only sampled at most once in a given year, but a minority (8.5% in 1996, 12.3% in 1997, 7.9% in 1998, 15% in 1999, 26% in 2000, and 31% in 2001) were sampled on two or more occasions in a single year. All farms are sampled at least once every 2 years. Data are insufficient to systematically analyze variation at sites.

The mean prevalence of IPNV was calculated for classes of samples, e.g., marine salmon farms in Shetland in 1996. (Prevalence was the proportion of samples that were IPNV positive for the class of site examined.) The use of prevalence removed the bias associated with different sampling frequencies. Confidence intervals were determined from the probability values for which distribution intercepts with observations, for the number of observations available. For a more formal test of when regional means deviated significantly from the overall mean, we used the χ^2^ test, because the data were binomially distributed. Site samples were not fully independent, but most sites were not sampled more than once in a year and thus were semi-independent. Clumping of samples could also have occurred for sites within hydrodynamically well-mixed environments (e.g., farms in the same sea loch).

Regional patterns exist for IPN; Shetland has a particularly serious problem (*16*). Regional variation in IPN-induced deaths is also apparent in Norway (*12*). Regional prevalence patterns are therefore examined by separating out salmon production sites into regions based on a United Kingdom ordnance survey 100-km^2^ grid. The regions identified are Shetland, Orkney, the Outer Hebrides, and mainland Scotland. Mainland Scotland was divided into northern and southern regions by using the 800-km north line of the United Kingdom ordnance survey’s national grid system (Figure 1). The interannual changes in IPNV prevalence have been examined at salmon production sites within the regions identified above.

## Multilevel Model

Both space and time play roles in determining variance in the observations. Simple multilevel models of the variance in the observations allow determination of the relative contributions of these components. These models, based on normal or binomial assumptions, were constructed by using the package MLwiN (*21*). The model was constructed with binomial variance distributed according to a logistic function IPNV = [1 + exp(–β)]^–1^. This model determined a mean β = –1.138 (±0.517), which gave a mean prevalence of 0.242 (24%). The logistic function was used because it varies from 0 to 1 and therefore covers the range of situations when IPNV is absent to situations when it is present in all samples within a group. The model was then used to determine the contributions of the different levels in the model to the variance about this mean.

The highest level of the model is the split between saltwater and freshwater environments. These salmon production sites are fundamentally different in nature (*3*), with different age classes of fish and routes of infection. Inclusion does add uncertainty to the model’s output because environment has only two classes. However, because saltwater and freshwater sites are related at other levels in different ways, this split in variance is explicitly modeled, rather than added as a fixed effect. The second level is the region; at this level, different climates, environments, and local organizations (e.g., the Orkney Fish Farmers Association) exist. The third level at which variance is resolved is that of year: because IPNV’s prevalence is increasing, prevalence changes over time. Seasonal data are also included because most IPNV cases occur in summer. Seasonal effect was analyzed by using three seasons of 4 months’ duration (January–April, May–August, and September–December) because individual months in winter are often poorly sampled.

## Results

Mean IPNV prevalence was determined by region, and a χ^2^ test of deviation from the overall Scottish mean was conducted (Table 1). High IPNV levels stood out in Shetland for both freshwater and seawater sites. Northern mainland Scotland and the Outer Hebrides showed a particularly low relative prevalence of IPNV in the marine sites and marginally significant low prevalence in freshwater. Orkney also had a low prevalence of IPNV in freshwater; however, because we obtained fewer data from Orkney, this finding was not significant.

**Table 1:** Mean prevalence of infectious pancreatic necrosis virus by region for all 1996–2001 data, and χ^2^ probability that prevalence is significantly different from the all-Scotland mean

Region	Saltwater		Freshwater	
	% Prevalence	χ^2^ p	% Prevalence	χ^2^p
All Scotland	49.6	N/A	10.6	N/A
North	41.1	0.001	5.5	0.1
South	55.5	–	11.1	–
Shetland	77.0	0.001	35.3	0.001
Orkney	48.5	–	6.7	–
Outer Hebrides	31.3	0.001	6.7	0.1

**Figure 2:**
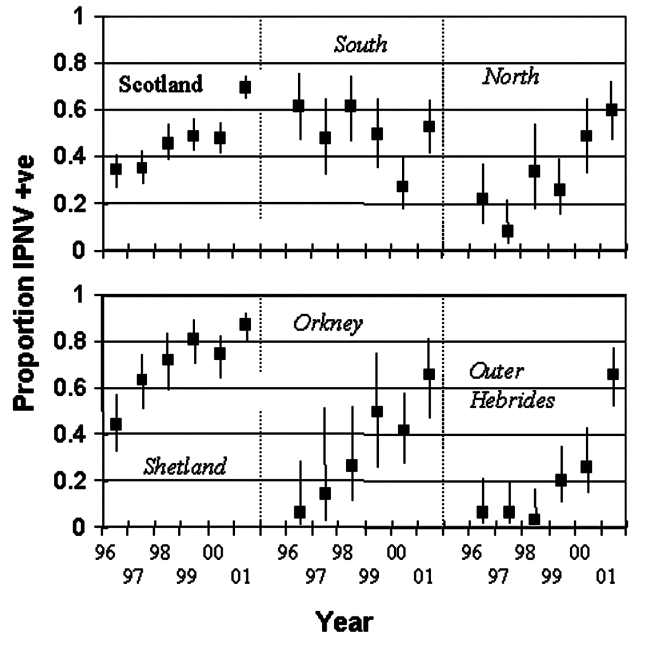
Prevalence of infectious pancreatic necrosis virus in Scottish marine salmon production sites by region and year. The regions are northern mainland Scotland, southern mainland Scotland, Shetland, Orkney, and the Outer Hebrides. Bars show 95% confidence intervals.

In marine sites, IPNV prevalence showed different temporal patterns in different regions (Figure 2). In most areas, IPNV prevalence appeared to have increased from 1996 to 2001 at approximately 10% per year (Table 2); the trends in these regions were not significantly different from each other or from the national mean. Analysis methods changed slightly in 2001; however, the trend of increasing prevalence was apparent throughout the entire 1996–2001 period. In southern mainland Scotland, IPNV prevalence was not statistically significant. If anything, it declined, although variation occurred from year to year. Linear increase in IPNV prevalence in Shetland became difficult to sustain once prevalence exceeded 80% in 1999, so the increase reached a saturation point (Figure 2).

**Table 2:** Trends in annual average prevalence of infectious pancreatic necrosis virus for all Scotland and by regions with regression 90% confidence intervals, probability, and r^2a^

Region	% Annual increase	90% CI	p	r^2^
Marine sites				
All Scotland	6.2	±3.1	0.012	0.82
South Mainland	-3.4	±6.2	0.306	0.26
North Mainland	8.7	±5.4	0.026	0.75
Shetland	7.4	±3.7	0.014	0.82
Orkney	11.5	±3.7	0.003	0.91
Outer Hebrides	10.6	±7.1	0.013	0.71
Freshwater Sites				
All Scotland	2.0	±1.5	0.051	0.66
South Mainland	1.9	±1.3	0.039	0.69
North Mainland	0.4	±1.9	0.663	0.05
Shetland	6.5	±3.9	0.026	0.75
Orkney	2.4	±2.3	0.097	0.54
Outer Hebrides	3.0	±2.2	0.041	0.69

^a^IPNV, infectious pancreatic necrosis virus; CI, confidence intervals; p*,* probability; r^2^, coefficient of determination for bivariate analysis.

**Figure 3:**
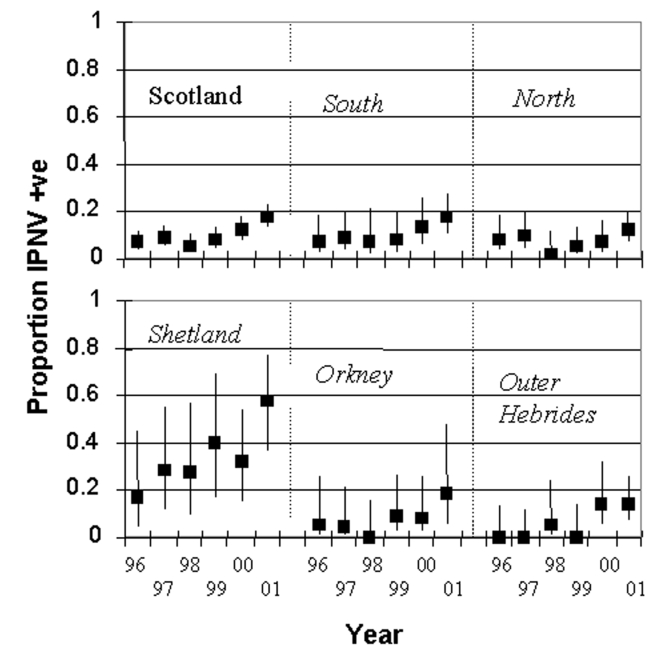
Prevalence of infectious pancreatic necrosis virus in Scottish freshwater salmon production sites by region and year. The regions are northern mainland Scotland, southern mainland Scotland, Shetland, Orkney, and the Outer Hebrides. Bars show 95% confidence intervals.

In freshwater production sites, interannual variation in IPNV prevalence increased approximately 2% to 3% per year (Figure 3**,** Table 2). However, in Shetland, the increase was faster at 6.5% year, which is significantly different from other regressions at 90% confidence. The analysis used has not changed for freshwater since 1993. In northern Scotland, no significant increase has occurred, although since 1999, prevalence has increased. As noted earlier, prevalence of IPNV in Shetland in freshwater is higher than in other areas and is increasing more rapidly.

**Figure 4:**
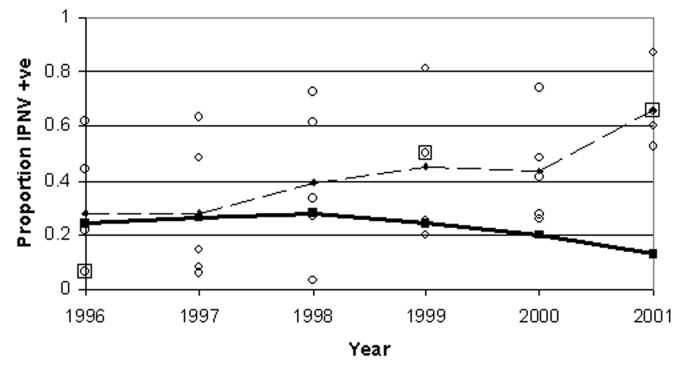
Mean of regional annual mean prevalence of infectious pancreatic necrosis virus in marine salmon farms (thin dashed line) and standard deviation of regional annual means (thick solid line), by year. Individual marine regional annual means are shown by circles (where two circles overlay, a large square is added). Standard deviation has fallen at an increasing rate, in spite of increasing mean regional prevalence.

Differences between regions have decreased in recent years in spite of increasing prevalence (Figure 4). The regions are becoming less distinct with respect to IPNV prevalence as the IPNV-positive regions converge.

The multilevel model has been used to determine how much different components contribute to the variation in model results from the overall mean of 24% (Table 3). The contribution is distributed, as expected from the observations described earlier. Most variance is accounted for at level 2, between regions. A substantial amount of variance is at the top level between saltwater and freshwater environments, but, because only two categories exist, the degree of this variance is uncertain. Since slightly more sensitive methods were used to sample IPNV in freshwater than in saltwater, this difference may be underestimated. A similar amount of variance is accounted for at the interannual level but with less uncertainty. Very little variation is due to season.

**Table 3:** Variance in the error terms obtained in a multilevel model with binomial assumptions regarding infectious pancreatic necrosis virus distribution

Level of variance	Standard error	Component
1	0.333 ± 0.474	Saltwater/freshwater
2	0.582 ± 0.341	Region
3	0.362 ± 0.111	Year
4	0.042 ± 0.055	Season

## Discussion

Our analysis systematically shows that variation exists in the distribution of IPNV in Scotland and allows a detailed picture of changes in distribution to be derived. The emergence of IPNV has been documented, with IPNV prevalence increasing from very low levels to high levels, even to near ubiquity in Shetland salmon farms. IPNV prevalence has typically increased annually by 10% in saltwater and by 2% to 3% in freshwater. In freshwater in Shetland, the annual increase was 6.5%.

In spite of IPNV’s emergence in most of Scotland, the virus’s prevalence did not change significantly in southern areas; if anything, it declined. The reasons for this are unclear, but the pattern suggests that controlling the emergence of IPNV may be possible. In Sweden, IPNV is controlled by culling, and the virus is officially absent from that country in spite of very high levels in neighboring Norway (*11*). Notably, in 1999 or 2000 in many areas of Scotland, marine IPNV prevalence declined, or at least increased less rapidly. This decline followed a period of fallowing, which in turn followed widespread culling to control the outbreak of infectious salmon anemia of 1998–99 (*22*,*4*).

Multilevel modeling shows that the regional pattern dominates the structure of variation in IPNV’s prevalence in salmon farms, overshadowing even the difference between marine and freshwater sites, although this could be slightly underestimated. The strength of this regional pattern is due to the extremely high prevalence of IPNV in Shetland and its low prevalence in the Outer Hebrides in both freshwater and saltwater sites.

The regional variation is in decline for marine salmon farms, however. As IPNV approaches ubiquity in Shetland, the virus has reached the saturation point; in many other areas, the increase remains rapid. In the Outer Hebrides, where prevalence was lowest, the increase has been the most rapid. As a result, IPNV prevalence levels are converging, and the standard deviation among regional means has declined. As the regions have become increasingly similar, regional differentiation in control policy becomes less useful.

Our analysis is not intended to describe the absolute prevalence of IPNV in Scotland. Detection limits (*9*,*10*) and, on occasion, small numbers of fish (*19*) may mean that infection may not always be detected at an infected site; thus, the true prevalence of IPNV within samples is probably higher than reported. Uncertainty in test results is also a fundamental limitation (*10*) and would apply to the results of a survey specifically designed to determine viral prevalence as much as it applies to these monitoring data. Sample site selection is not random but is based on the need to monitor for IPNV, which may also lead to biases. However, with a centralized organization that collects data by standardized methods, trends and regional variation may be analyzed in relative terms.

Improved analysis methods may have contributed to increases in reported IPNV prevalence in marine water in 2001; however, this increase continues a trend and reflects an increase in clinical IPN cases (*11*,*15*). Increased experience per se is unlikely to be a factor in increased detection during the study period since the FRS Marine Laboratory has been sampling for IPNV since the 1970s (*18*) and has contributed to the development of standard tests (*9*). Moreover, prevalence shows different temporal patterns in different areas, in spite of centrally controlled methods and personnel. These increases therefore cannot be explained in terms of improvements in methods, nor can differences in methodology be used to explain regional differences in prevalence. In addition, sampling methods used in freshwater may be slightly more sensitive than those used for marine sites, which could mean the difference in prevalence between fresh and marine waters is even larger than reported here**.** Because IPNV has a wide variety of host species (*7*,*13*), persistent carriers among recovered hosts (*23*), and the ability to be carried by piscivorous bird vectors (*24*), the virus is difficult to eradicate, once established. Given IPNV prevalence increases of approximately 10% per year, effective control would have to be reestablished very soon if IPNV is not to become as ubiquitous in most areas as it is in Shetland. Owing to limitations in detection methods, IPNV prevalence in samples may be even higher than reported (*10*). IPNV prevalence has been held steady in southern areas of Scotland, indicating that an increase is not inevitable; Sweden has maintained IPNV-free status. In freshwater sites in Scotland, IPNV is less prevalent, and the prevalence is increasing at lower rates; therefore, control can likely be reasserted in freshwater.

IPNV is one of a series of emerging pathogens causing serious economic damage to aquaculture in Scotland (*6*,*22*) and around the world (*1*,*2*). Although data collected from programs to monitor these pathogens are not always statistically unbiased, they form a valuable resource on emerging and established (*8*) diseases. As further fish species are cultivated (e.g., cod *Gadus morhua* [*3,7*]), such data are required to design a sustainable industry.
